# Clinico-Microbiological Profile and Treatment Outcome of Infectious Scleritis: Experience from a Tertiary Eye Care Center of India

**DOI:** 10.1155/2012/753560

**Published:** 2011-11-20

**Authors:** Srikant Kumar Sahu, Sujata Das, Savitri Sharma, Kalyani Sahu

**Affiliations:** ^1^Cornea and Anterior Segment Service, L V Prasad Eye Institute, Bhubaneswar 751024, India; ^2^Ocular Microbiology Service, L V Prasad Eye Institute, Bhubaneswar 751024, India

## Abstract

Medical and microbiology records of seventeen patients (17 eyes), diagnosed as scleritis of infectious origin were reviewed; to study clinical features, predisposing risk factors, microbiologic profile and treatment outcome of infectious scleritis. The mean patient age was 52.3 ± 19.75 years. Twelve patients (70.6%) had history of trauma/prior surgery. Isolated organisms included *Staphylococcus* species (spp) (*n* = 5), Fungus (*n* = 4), *Nocardia* spp (*n* = 3), two each of atypical *Mycobacterium* spp and *Streptococcus pneumoniae* and one *Pseudomonas aeruginosa*. Treatment included intensive topical antimicrobial in all eyes and systemic medication in 15 (88.2%) patients; surgical exploration was needed for 13 (76.5%) patients and scleral patch graft was done in four (23.5%) patients. Lesions resolved in all patients and none required evisceration. The presenting log MAR visual acuity of 1.77 ± 1.40 and improved to 0.99 ± 0.91. (*P* ≤ 0.039) after treatment with a mean follow up of 22.57 ± 19.53 weeks. A microbiological confirmation, appropriate medical and/or surgical intervention has a good tectonic and visual outcome.

## 1. Introduction

Infectious scleritis presents as an ulcerated or nonulcerated, inflamed scleral nodule [1]. It accounts for 5–10% of all cases of scleritis [2–5]. But the presenting picture of infectious scleritis may not differ too much from immune-mediated scleritis [6–10]. Approximately 40–90% of immune mediated scleritis have an associated systemic vascular disease [3, 4, 6, 10]. While systemic treatment with corticosteroids or immunosuppressant benefits immune-mediated scleritis, it worsens the infectious scleritis. Hence it is imperative to differentiate between two conditions. 

Many authors have reported infection by *Pseudomonas aeruginosa* and fungus as the most common causative organism [1, 11–13]. Pterygium surgery with beta radiation or application of mitomycin C has been identified as a common risk factor for infectious scleritis [11–13]. 

The clinical outcome is generally poor and most cases required evisceration in the many series [8, 9, 14]. Systemic and topical medication combined with early surgical intervention have improved the anatomical success, but not the visual outcome in two other series [1, 12]. 

Infectious scleritis is a rare entity; hence it is not suspected at the initial presentation resulting in delayed diagnosis and treatment. In this communication, we describe the predisposing factors, clinical features, etiology, and treatment outcome of infectious scleritis.

## 2. Material and Method

We retrospectively reviewed the medical and microbiological records of all patients with microbiologically proven infectious scleritis examined at Cornea Services of L V Prasad Eye Institute Bhubaneswar from November 2006 to August 2009. At presentation, all patients had received a detailed ophthalmic examination in the office. Patients with ulcerative lesions had received a scraping with a no. 15 surgical blade on a Bard Parker handle from the base and active edges of the ulcer under topical anesthesia in the office, and all nonulcerative nodular abscesses had received scleral scraping in the operating room under peribulbar anesthesia. In the later case, the base of the lesion was scrapped after dissecting the conjunctiva and deroofing of the nodular lesion. Materials collected from the lesions were smeared on glass slides and stained with Potassium hydroxide + Calcoflour white stain, Grams stain, Zeihl Neelsen stain using 20% H_2_SO_4_ or modified Zeihl Neelsen stain using 1% H_2_SO_4_. Acid fast staining was usually done if Grams stain smear was negative, but the lesions were strongly suspicious of microbial origin. The exudates from the lesion were cultured on blood agar, chocolate agar, Sabouraud's dextrose agar (SDA), Brain-heart infusion broth and non-nutrient agar with an *Escherichia coli* overlay. All media were incubated at 37°C except SDA; this was incubated at 27°C. Significant growth was defined as confluent growth on solid media, and/or there was growth of the same organism on more than one medium, and/or growth in one medium was accompanied by presence of similar organism in smears. All bacteria and fungi grown were identified as per standard protocol, and bacteria were tested for antibiotic susceptibility by Kirby-Bauer disc diffusion method. 

The initial therapy either was based on results of smear or when smear was negative, an empirical treatment with topical antibiotic (mostly gatifloxacin 0.3% and amikacin 2.5%) along with systemic gatifloxacin ((400 mg) twice daily) was given. The treatment was modified, if needed, based on final culture and sensitivity report. Surgical debridement was done should the scleral ulceration area extend locally or progressed to form a subconjunctival abscess at another site away from the main lesion.

The collected retrospective data included patient demography (age, gender, occupation), disease history (onset, course, predisposing factors), clinical feature, type of organisms, antibiotic susceptibility, treatment given, and the outcome.

## 3. Results

We included 17 patients (17 eyes) of infectious scleritis between November 2006 and August 2009. The inclusion criteria were presentation with a scleral ulcer and/or abscess and an organism isolated microbiologically. This included 7 women and 10 men; the age ranged from 13 years to 75 years (mean 52.3 ± 19.75 years, median 55 years). (Table 1) The mean followup was 22.57 ± 19.53 weeks (range of 3–89 weeks). 

The most common predisposing factor was an ocular surgery [*n* = 9; 52.9%. 95% (CI), 29.27–76.73]. The surgery included cataract in seven eyes, scleral buckle in one eye, and trabeculectomy in one eye. The interval from surgery to diagnosis of infectious scleritis ranged from one week to four years. Four patients had injury with organic material like wood and mud three weeks to seven months prior to presentation (mean 37 ± 33.04 days; median 21 days). Fifteen of 17 patients were using topical corticosteroids and two patients were using oral corticosteroids at the time of reporting to us. Only one patient was diabetic in this series.

**Figure 1:**
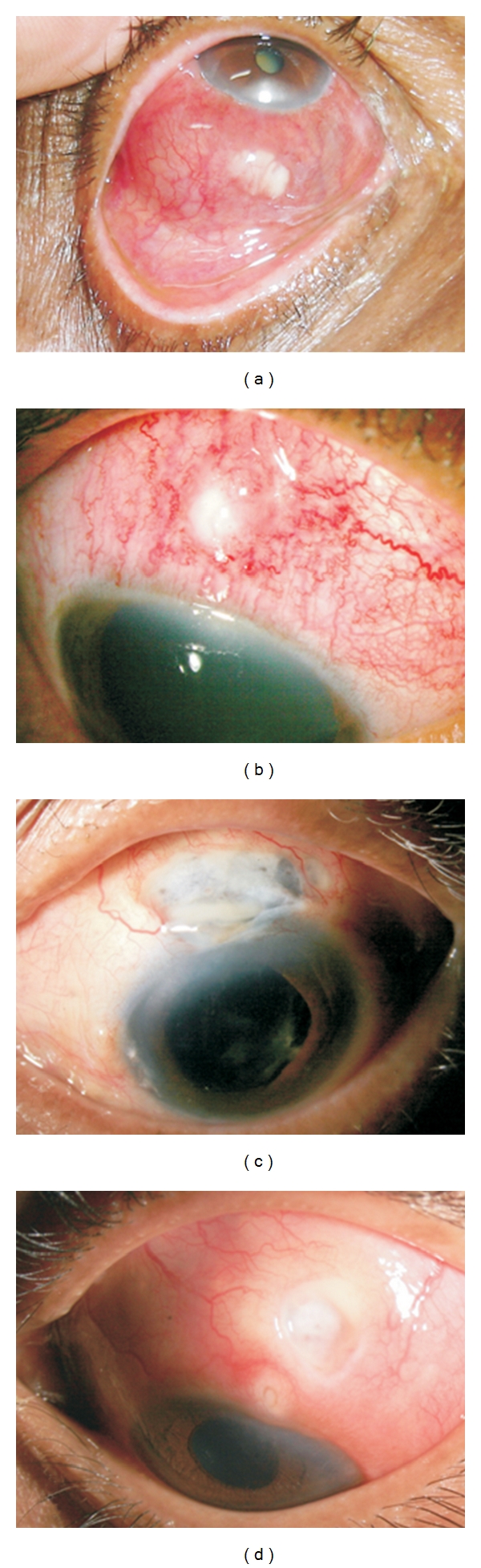
Slit lamp picture depicting different clinical presentation. (a) Case no. 2: multiple scleral abscess. (b) Case no.12: single scleral abscess. (c) Case no. 4: necrotic ulcer (post cataract surgery). (d) Case no. 3: two punched out ulcers.

**Figure 2:**
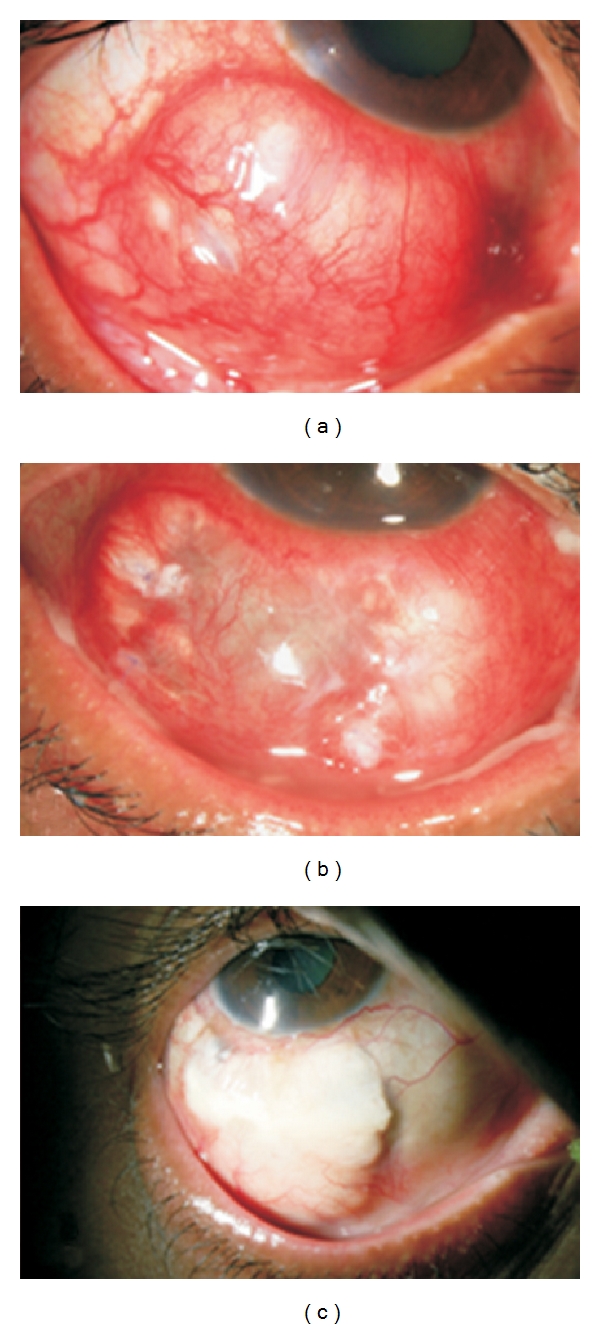
Slit lamp picture of Case no. 10. (a) At Presentation showing multiple abscesses and ulcer. (b) Three weeks after initiation of treatment (surgical exploration and medical management), there is a uveal show. (c) Final outcome showing vascularized scleral graft (four months after scleral patch graft).

The symptoms present in all patients were redness, pain, and watering in the affected eye. The presenting visual acuity varied from hand motions (HM) to a normal vision of 20/20. Twelve patients (70.54%) presented with a vision less than 20/40. Unifocal or multifocal scleral abscess was seen in six patients (35.39%) (Figures 1(a) and 1(b)). Characteristically, the abscesses presented as yellowish nodules under the intact conjunctival epithelium. Scleral ulceration and necrosis was seen in eight patients (47.05%) (Figures 1(c) and 1(d)). Necrosis around the incisional area was seen in all seven patients who had received cataract surgery earlier. Three eyes (17.34%) had both ulceration and abscess (Figure 2(a)). 

The culture grew a variety of organisms. They included *Staphylococcus* species (*n* = 5; 29.41%), fungus (*n* = 4; 23.52%), *Nocardia* species (*n* = 3; 17.6%), *Streptococcus pneumoniae* (*n* = 2), atypical* Mycobacterium *(*n* = 2), and *Pseudomonas aeruginosa *(*n* = 1). One of the four Staphylococcus was methicillin resistant (no. 7). Of the four fungi isolated, two could not be identified, one belonged to Fusarium and Paecilomyces species each.

The results of antibiotic susceptibility testing for bacterial isolates (Kirby Bauer disc diffusion method) are given in Table 2. *Staphylococcal *and *streptococcal *cases were treated with fortified Cefazolin (50 mg/mL) and a flouroquinolone (ciprofloxacin 0.3%/gatifloxacin 0.3%) along with systemic fluoroquinolones (ciprofloxacin 500 mg/gatifloxacin 400 mg twice daily). Patient no. 7 was treated only with topical ciprofloxacin. Fungal scleritis was treated with topical natamycin 5% and systemically either itraconazole (100 mg) or ketoconazole (200 mg) two times daily. *Nocardia *and atypical* Mycobacterium* scleritis were treated primarily with topical fortified amikacin (25 mg/mL). Systemic trimethoprim (160 mg) sulphamethoxazole (800 mg) (TMX-SMZ) combination was used in patients of *Nocardia* scleritis only.

Thirteen of the 17 patients underwent wound debridement. During the surgical debridement, it was noticed that the area actually involved was generally much larger than initially seen under slit lamp. N-Butyl Cyanoacrylate glue was used in two patients as there was a limbal perforation of less than 1 mm × 1 mm in size. Five patients received scleral patch graft; as primary procedure in one (patient no. 4, Figure 1(c)), and the remaining four eyes, 2 days to one month after initiation of medical treatment. Seven eyes needed multiple surgical interventions.

Oral corticosteroid (1 mg/kg of body weight) was used in 5 eyes with bacterial scleritis two days after antibacterial treatment. In case no. 4, scleral patch graft was done as the primary procedure, later fungal filaments were identified from smear; so corticosteroids were started only after two weeks when recurrence was not noticed. Intraocular antibiotic was used in three patients suspected to have endophthalmitis. All of them presented scleral infection after cataract surgery.

Resolution was defined as absence of symptoms, congestion, or active infiltrate. All the patients in this series responded to treatment; 13(76.47%) patients had only scarring with no or minimal uveal show; one patient required scleral patch graft (Patient no. 10 Figures 2(a), 2(b), and 2(c)) one month after initiation of treatment. In the patients where scleral patch graft was done, success was defined as no evidence of graft or surrounding infiltrate and vascularization of the graft. All five grafts were healthy at the last visit. 

**Figure 3:**
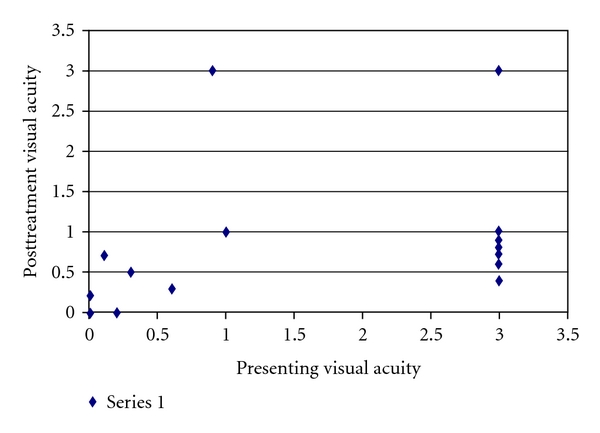
Comparision of the presenting visual acuity with posttreatment logMAR visual acuity of all patients.

The mean presenting and post treatment logMAR visual acuity was 1.77 ± 1.40 and 0.99 ± 0.91. (*P* ≤ 0.039) (Figure 3). The vision improved by greater than 2 lines in 8 of 12 patients who presented with a visual acuity of <20/40 patients. 

The primary cause of decreased vision after resolution of infection was cataract and corneal scar. Total choroidal detachment developed in two patients and was treated with tapering oral corticosteroids. One of the two patients had atypical* Mycobacteria* infection. This 19-year-old lady also developed a corneal abscess after seven months of treatment. It resolved with topical fortified amikacin. 

## 4. Discussion

Scleritis may represent a diagnostic challenge and is often associated with life-threatening systemic disease (in this series, only one patient had diabetes mellitus though) and vision-threatening ocular complications [15]. 

Scleral infection from *Pseudomonas*, *Staphylococcus*, or Herpes zoster virus can cause necrotizing scleritis, which is clinically identical to systemic autoimmune disease [6]. Unusual organisms like *Nocardia, Acanthamoeba, *atypical* Mycobacteria*, *Mycobacterium tuberculosis*, and *Listeria monocytogenes* are known to cause scleritis [1, 16–18]. Often the diagnosis of an associated infection or systemic condition dictates therapy. Systemic vasculitis typically requires systemic corticosteroid or immunosuppressive drugs [2, 3]. An early and definitive diagnosis helps in treatment of the condition and has better outcome.

Patients with a history of prior ocular surgery or trauma and presenting with a scleral abscess or ulceration and necrosis should arouse the suspicion of infectious origin. The surgeries include pterygium, cataract, scleral buckling, and strabismus surgery [10–12, 18]. In our series, cataract surgery was the predisposing factor in seven eyes and trauma with organic material was present in four eyes.

Corticosteroids, given before infection control, in an infectious scleritis worsen the condition by inhibiting release of lysosomal enzyme. In this cohort, 15 of 17 patients were on topical corticosteroids at presentation to us. It is unknown whether this aggravated the infection or decreased the immunity for secondary infection to occur.

Bacterial infectious scleritis is more common than other infectious scleritis. *P. aeruginosa *has been the often reported infecting organism [10, 12, 13, 15]. It was reported in over 50% eyes from Taiwan following pterygium surgeries [12, 13]. In our cohort, however, *Staphylococcus* infection (24.41%) and cataract were the most common surgery (41.17%). In our series, only one of 17 patients had *P. aeruginosa* infection. 

One of our coauthors has reported fungal infectious scleritis in over one of third patients and high incidence of *Nocardia *infection from Hyderabad (south central India) [1]. In this series, we detected fungus in close to a quarter of patients and *Nocardia* in close to 20 percent. High incidence of fungus in both series can be attributed to the hot and humid climate and the enormous amount of fungal spore prevalent in the environment [19]. Two of the three patients with *Nocardia* scleritis could not recollect any history of injury although one of them was an agriculturist. All three patients with *Nocardia *scleritis required multiple explorations of the abscess, and one patient (no. 10) (Figures 2(a), 2(b), and 2(c)) required a scleral patch graft after resolution of the active infection. 

In addition to topical medications, systemic antibiotic or antifungal was required for 15 patients. Systemic corticosteroids were used in patients with bacterial infection (*n* = 5), in eyes that developed choroidal detachment (*n* = 2), and in the patient who developed exophthalmous (no. 12). 

Pyogenic infections of the sclera are often difficult to eradicate because of poor antimicrobial penetration into the avascular necrotic sclera; combination of surgery such as abscess exploration and systemic and topical antimicrobial therapy yields superior results [1, 13, 20, 21]. Surgical debridement not only facilitates penetration of antibiotic but also debulks the infected scleral tissue. 

We do agree with Lin et al. who suggested mandatory surgical exploration of the abscess that does not respond to initial medication to increase the penetration of antibiotic [13]. Raber et al. reported a “tunnel lesion” on histopathological finding in cases of scleral ulcer [22]. Our experience is similar to Lin et al. who also described the tunnel lesion and recommended the need of careful exploration to avoid residual infection [13]. Two (no. 2, 14) of our 7 patients in this series required multiple surgical intervention and both of them had “tunnel lesion”. 

All 17 patients in this series achieved anatomical success. Fourteen of 17 patients (82.35%) regained useful vision (defined as vision ≥20/200). The causes of poor vision in the remaining three eyes were glaucomatous optic atrophy (no. 11), chronic retinal detachment (no. 13), and fibrotic membrane in the pupillary area (no. 17). In an earlier series by one of our coauthors, one-third of patients had regained useful vision, but three of 21 eyes were eviscerated and one eye became phthisical [1]. Eleven of the 18 eyes in Hsiao et al. series retained useful vision, three had poor vision and four had to be eviscerated [12]. None of our patients in this series required evisceration. 

Associated uveitis is not uncommon. (66% in Su et al. series) These inflammations lead to formation of pupillary membrane, cataract, and endophthalmitis [10]. Most patients in our series had low-grade inflammation in the anterior chamber at presentation; severe intraocular inflammation occurred in five patients subsequently. Cataract was the most common sequel in our series (*n* = 3). Four patients who had extension to cornea resolved with corneal scar. 

Our series has all weaknesses inherent to all retrospective studies. Our institution is a tertiary care referral center, so there is a potential for bias towards more unusual or difficult-to-control diseases. Nonetheless, we have demonstrated that a variety of organism can cause scleritis. An early clinical suspicion of infectious origin, identification of the infecting organism, knowledge of antibiotic susceptibility, and institution of appropriate medical and/or surgical therapy could have good tectonic and visual outcome.

## Figures and Tables

**Table 1:** Patient demographic, clinical, and microbiological feature, management, and outcome.

Sl. no.	Age	Sex	Eye	History	Duration of symptoms (in days)	Presenting visual acuity	Clinical signs	Organism	Treatment			Final visual acuity	Remarks	Outcome
									Topical	Systemic	Surgical			
1.	72	M	OS	Cataract surgery	45	CF 2 m	Necrosis, corneal infiltrate	*S. aureus*	F. cefazolin, ciprofloxacin	Ciprofloxacin	TA + BCl	20/70	Corneal scar	Resolved
2.	65	M	OD	Nil	60	20/40	Abscess	*Nocardia spp.*	F. amikacin, gatifloxacin	TMX-SMZ	Exploration-2	20/60p	Cataract	Resolved
3.	19	F	OS	Nil	150	20/25	Punched out ulcer	*Atypical mycobacterium*	F. amikacin, ciprofloxacin	TMX-SMZ, prednisolone	Nil	20/30	Corneal infiltrate, choroidal	Resolved
4.	72	M	OS	Cataract surgery	45	CF 2 m	Necrosis	*Fusarium spp.*	Natamycin, gatifloxacin, cyclosporine	Itraconazole	SPG	20/120	Graft vascularized	Resolved
5.	35	F	OD	Injury (with stick)	15	CF 2 m	Abscess, corneal infiltrate	Fungus	Natamycin, gatifloxacin	Itraconazole	Exploration, Intracameral ampho- B	20/160	Corneal scar and cataract	Resolved
6.	66	M	OD	Cataract surgery	9	HM	Scleral necrosis, exudate	*S. pneumoniae*	F. cefazolin, gatifloxacin, betamethasone	Lizolidine, predinisolone	SPG + IOAB	20/60	Graft vascularized	Resolved
7.	49	M	OD	Nil	120	20/20	Ulcer, abscess, thinning	*Staphylococcus spp.*	Ciprofloxacin, prednisolone	Prednisolone	Nil	20/20	Nil	Resolved
8.	50	M	OS	Cataract surgery	7	HM	Necrosis	*S. aureus*	F. cefazolin, ciprofloxacin	Gatifloxacin	SPG + Vitreous biopsy + IOAB	20/100	Graft vascularized, corneal scar	Resolved
9.	74	F	OS	Injury (with mud)	15	HM	Abscess	*P. aeruginosa*	F. amikacin, Ciprofloxacin, Prednisolone	Ciprofloxacin, prednisolone	Exploration	20/200	Cataract, choroidal	Resolved
10.	50	F	OD	Injury (with twig)	75	20/30	Chemosis, abscess	*Nocardia spp.*	F. amikacin, ciprofloxacin	Ciprofloxacin	Exploration SPG + AMG	20/20	Graft vascularized	Resolved
11.	13	M	OD	Trabeculectomy	30	CF 1 m	Abscess	*S. aureus*	F. cefazolin, gatifloxacin	Cefadroxil	Nil	CF 1 m	Glaucoma, optic atrophy	Resolved
12.	21	M	OS	Thyroid Ophthalmoplegia	Not available	20/20	Abscess	*S. pneumoniae*	F. gentamicin, chloramphenicol	Prednisolone	Nil	20/30	Nil	Resolved
13.	53	M	OS	Retinal detachment surgery (buckle)	60	CF 1 m	Ulcer, abscess	*Atypical mycobacterium*	F. amikacin, gatifloxacin	Gatifloxacin	BB Removal	HM	Elevated Granuloma	Resolved
14.	55	F	OS	Nil	60	20/200	Thinning, ulcer, abscess	*Nocardia spp.*	F. amikacin, ciprofloxacin	Ciprofloxacin, TMX-SMZ	Exploration-3	20/50	Nil	Resolved
15.	75	F	OD	Cataract surgery	60	HM	Necrosis	Fungus	Natamycin	Itraconazole	TA + BCL	20/50	Corneal scar	Resolved
16.	65	F	OS	Cataract surgery	7	20/70	Necrosis, corneal infiltrate	*S. aureus*	F. cefazolin, gatifloxacin, prednisolone	Ciprofloxacin	Wound repair + IOAB	20/40	Membrane over IOL	Resolved
17.	55	M	OS	Cataract surgery	30	20/160	Necrosis	*Paecilomyces spp.*	Natamycin	Ketoconazole	SPG	PL+	Pupillary membrane	Resolved

**Table 2:** Results of antibiotic susceptibility testing for bacterial isolates (Kirby Bauer disc diffusion method).

Sl. no	Patient no. (from Table 1)	Name of the bacteria	Antibiotics						
			Chlo	Cefa	Vanco	Cipro	Gati	Oflo	Amik
1	1	*S. aureus*	S	ND	ND	S	S	ND	ND
2	2	*Nocardia* sp.	S	S	ND	S	S	ND	S
3	3	Atypical mycobacteria	S	S	S	S	S	ND	S
4	6	*S. pneumoniae*	S	S	S	S	S	S	ND
5	7	*Staphylococcus* sp.	S	S	S	R	S	I	ND
6	8	*S. aureus*	S	S	S	I	I	I	ND
7	9	*P. aeruginosa*	R	ND	ND	S	S	S	S
8	10	*Nocardia* sp.	R	R	R	S	S	S	S
9	11	*S. aureus*	R	S	S	R	I	I	ND
10	12	*S. pneumoniae*	S	S	S	S	S	S	ND
11	13	Atypical mycobacteria	R	R	R	R	R	R	S
12	14	*Nocardia* sp.	S	R	S	R	S	R	S
13	16	*S. aureus*	S	S	S	I	S	I	ND

Chlo: Chloramphenicol, Cefa: Cefazolin, Vanco: Vancomycin, Cipro: Ciprofloxacin, Gati: Gatifloxacin, Oflox: Ofloxacin, Amik: Amikacin, S: Sensitive, I: Intermediate, R: Resistant, ND: Not done.
